# Invest in youth led efforts for gender equality and pandemic preparedness

**DOI:** 10.1136/bmj.p2861

**Published:** 2023-12-05

**Authors:** Merette Khalil, Cyubahiro Karangwa Verite, Shakira Choonara

**Affiliations:** 1 Women in Global Health Egypt, Cairo, Egypt; 2 Medical Students Association of Rwanda, Kigali, Rwanda; 3 Lancet Commission on Adolescent Health and Wellbeing, Johannesburg, South Africa

## Abstract

Young leaders are critical for research, programme design, and advocacy

Women, people aged under 30, and marginalised populations, especially from low and middle income countries (LMICs), continue to face barriers to participation in global health governance systems. As a result, these voices often go unheard and are not included in decision making processes related to health. As the BMJ collection on gender equality and pandemic response shows (www.bmj.com/gender-and-pandemic-response **)**,^1^ traction is growing for recognising the importance of applying a gender lens within pandemic preparedness research and policy. However, adolescents (12-19 year olds) and youth (under 30s) remain missing within multistakeholder health emergency and disaster committees, from health system preparedness and responses at all levels (global, regional, national, and local), and within the research agenda.^2^

Covid-19 has essentially reversed gains in gender equality, widening socioeconomic, educational, and health inequalities for women and the world’s 1.8 billion young people (aged 10-24 years).^3^ ^4^ During the pandemic and its aftermath, women and girls carried an unequal burden of care, faced increased gender based violence, and endured severe disruptions to their health and educational services.^3^ According to Unesco, the pandemic risked more than 11 million girls not returning to school, with those in LMICs at particular risk.^5^ In Kenya, adolescent girls who remained out of school for six months because of the covid-19 lockdown had twice the risk of becoming pregnant and three times the risk of dropping out of school compared with their cohort graduating before to the outbreak.^6^ As a result of covid-19, 25% of youth in Mexico, Pakistan, South Africa, and Vietnam were not in education, employment, or training, and the World Bank estimated that global youth earnings contracted by 15% in 2020 and 12% in 2021, and predicted 10% loss on future earnings.^7^

The pandemic, along with the intensifying climate crisis, has also exacerbated mental health conditions, especially among young people.^8^ ^9^ Globally, one in seven adolescents experiences a mental health condition (around 80% of whom are in LMICs).^4^ The latest *World Mental Health Report* indicated a 25% increase in global rates of anxiety and depression in the first year of the pandemic, with further evidence confirming a higher prevalence and increased risk among youth, especially girls, those with disabilities, those living in emergency and conflict affected settings, and indigenous, sexual, and ethnic minorities.^10^ Moreover, a global survey on climate anxiety among young people found that more than 60% felt extremely worried and 84% moderately worried about climate change, with high correlation between anxiety and perceived inadequate governmental response to the climate crisis.^9^

The solution to reducing health and gender inequalities requires an intersectional and multisectoral approach that considers social, political, environmental, environmental, and structural factors.^2^ In planning for future health emergencies, whether the next pandemic or the climate crisis, it is crucial to ensure that those most directly affected (such as indigenous and displaced youth from LMICs) are engaged throughout the humanitarian responses cycles (from preparedness to recovery).^11^

Despite the detrimental effect of covid-19 on youth health and wellbeing, youth remain largely neglected within global political commitments on pandemic preparedness. At the 78th UN general assembly in September 2023, member states unanimously adopted the first political declaration on pandemic preparedness and response, which includes commitments to strengthen women’s meaningful participation, leadership, and decision making and to reduce the 24% gender pay gap in health.^12^ While, we applaud the recognition of gender issues within the political declaration, it does not focus on youth and their needs. Going forward there must be meaningful inclusion and engagement of young leaders in pandemic governance structures, ranging from the global Independent Panel for Pandemic Preparedness and Response and WHO’s pandemic accords to national health security committees.

It is not only the policy space where youth need to be included. In times of crisis it is also important to consider who is contributing to health research and who is being left out of clinical trials and author groups. For example, the BMJ collection on gender equality and pandemic response^1^ sets out a clear innovative and intentional commitment to foster collaborative and multistakeholder agenda setting, evidence generation, and action. The global collaboration brought together more than 500 stakeholders including researchers, donors, multilateral agencies, and civil society organisations to set priorities and implement a research agenda.^13^ However, it is unclear how many young researchers were authors or participated in the consultation and activities of the collaboration. Youth mainstreaming is just as important as gender mainstreaming.

In the areas of advocacy and programming, youth have shown commendable leadership and have served as innovators and implementers in support of the pandemic response. As an example, we were involved in a range of pandemic response activities at the local, national, and regional levels, including volunteering to track unavailability of personal protective equipment in South African public health facilities, providing public health expertise in the media—eg, national television, newspapers, and radio—leading covid-19 research on women and girls living with HIV in Rwanda, and supporting the development of hospital guidance on response, recovery, and resilience in the Eastern Mediterranean region. 

At the international level, Global Youth Mobilization, founded in 2020 by some of the world’s largest youth organisations, together with WHO and the UN Foundation, provided a collective global response to support youth led solutions to the effects of the covid-19 pandemic.^14^ Advocacy and fundraising by the collective contributed to $5m investment (£4m; €3.6m) in 471 youth led projects in 72 countries, directly engaging 600 000 young people and implementing activities reaching over 3.63 million community beneficiaries.^14^ Youth helped tackle misinformation during the covid-19 pandemic, including working in communities to build awareness and uptake of vaccinations in Micronesia^15^ and across the African continent through the African Centres for Disease Control’s Bingwa initiative.^16^

Evidence suggests that for every dollar spent investing in adolescent and youth wellbeing, including their agency and leadership, there is a 5-10-fold return on investment.^17^ Looking ahead the critical role young leaders play in health emergency research, programme design, implementation, and policy advocacy must be recognised and supported.^18^ Academics, policy makers, and emergency response leaders in health and other sectors should adopt and implement the global consensus statement on meaningful adolescent and youth engagement.^19^ They should also consider the five levels of meaningful youth engagement and their respective strategies where young people are informed, consulted, involved, collaborators, and empowered, throughout the emergency response.^20^ Emergency managers at all levels (facility, community, and policy) would benefit from using WHO’s recently published step-by-step implementation tool and five step checklist on empowering youth in health emergencies.^20^

Young people are rising to the challenge of emerging polycrises, including economic uncertainty, political instability, humanitarian events, and climate change. In recovering and rebuilding back better from the covid-19 pandemic, young people’s voices and leadership must be meaningfully and systematically integrated throughout pandemic and health emergencies research, response, and policy cycles. We believe doing so will contribute to better health and wellbeing outcomes for youth along with higher levels of civic engagement, accountability, and action.

## Notes

Provenance and peer review: Commissioned; externally peer reviewed.

This article is part of a collection proposed by the United Nations University and the University of the Western Cape and commissioned by The BMJ. The BMJ reviewed, edited, and made the decision to publish. Open access fees are funded by Bill and Melinda Gates Foundation and UN University-International Institute for Global Health.
